# Aberrant frontal lobe “U”-shaped association fibers in first-episode schizophrenia: A 7-Tesla Diffusion Imaging Study

**DOI:** 10.1016/j.nicl.2023.103367

**Published:** 2023-03-05

**Authors:** Jason Kai, Michael Mackinley, Ali R. Khan, Lena Palaniyappan

**Affiliations:** a Department of Medical Biophysics, Schulich School of Medicine & Dentistry, The University of Western Ontario, London, Ontario, Canada; b Robarts Research Institute, The University of Western Ontario, London, Ontario, Canada; c Lawson Health Research Institute, London, Ontario, Canada; d Douglas Mental Health University Institute, Department of Psychiatry, McGill University, Montreal, Quebec, Canada

**Keywords:** “U”-fibres, First-episode schizophrenia, Psychosis, Tractography, Diffusion MRI

## Abstract

Schizophrenia is believed to be a developmental disorder with one hypothesis suggesting that symptoms arise due to abnormal interactions (or disconnectivity) between different brain regions. While some major deep white matter pathways have been extensively studied (e.g. arcuate fasciculus), studies of short-ranged, “U”-shaped tracts have been limited in patients with schizophrenia, in part due to the sheer abundance of tracts present and due to the spatial variations across individuals that defy probabilistic characterization in the absence of reliable templates. In this study, we use diffusion magnetic resonance imaging (dMRI) to investigate frontal lobe superficial white matter that are present in the majority of study participants, comparing healthy controls and minimally treated patients with first-episode schizophrenia (<3 median days of lifetime treatment). Through group comparisons, 3 out of 63 frontal lobe “U”-shaped tracts were found to demonstrate localized aberrations affecting the microstructural tissue properties (via diffusion tensor metrics) in this early stage of disease. No associations were found in patients between aberrant segments of affected tracts and clinical or cognitive variables. Aberrations in the frontal lobe “U”-shaped tracts in early untreated stages of psychosis occur irrespective of symptom burden, and are distributed across critical functional networks associated with executive function and salience processing. While we limited the investigation to the frontal lobe, a framework has been developed to study such connections in other brain regions, enabling further extensive investigations jointly with the major deep white matter pathways.

## Highlights

• Identified aberrant frontal-lobe “U”-fibres in first-episode schizophrenia patients.
• Observed localized abnormalities along-tract associated withtissue microstructure.
• Changes to “U”-fibres occured irrespective of symptom severity.
• Proposed framework to track progression of diffusion measures in patients.

## 1 Introduction

Schizophrenia is a neuropsychiatric disorder with a diverse range of symptoms which can present differently amongst individuals [Stephan et al., 2009). Characterization of symptoms can be largely defined into three groups: (1) positive/psychotic (altered perception; e.g. delusions), (2) negative/deficit (reduced or lack of normal function; e.g. loss of motivation), or (3) cognitive (e.g. impaired attention or memory) (Jauhar et al., 2022, Rubinov and Bullmore, 2013). First proposed by Wernicke, it has been suggested that psychosis, commonly experienced by those diagnosed with schizophrenia, may arise from abnormal interactions resulting from disrupted brain connectivity (Fornito et al., 2012, Stephan et al., 2009, Wernicke, 1906), specifically between the prefrontal cortex and other brain regions (McIntosh et al., 2008). While many regions of the brain may be associated with schizophrenia, the frontal lobe is one of the most studied cortical regions with findings including physiological, morphological, and metabolic changes (Mubarik and Tohid, 2016).

Diffusion tensor imaging (DTI), a model commonly derived from diffusion magnetic resonance imaging (dMRI), can be used to evaluate quantitative changes in white matter and is sensitive to changes to the microstructural environment (Basser et al., 1994). In previous studies of psychosis, DTI has been mostly applied to study major white matter tracts connecting distant brain regions. The Schizophrenia Working Group of the Enhancing Neuroimaging Genetics through Meta-Analysis consortium (ENIGMA-Schizophrenia) studied DTI-derived measures in major white matter tracts, comparing differences between healthy controls and participants with schizophrenia (Kelly et al., 2018). In this study comparing over 4000 individuals, WM changes in patients with schizophrenia were found throughout the brain (Kelly et al., 2018). Regional abnormalities in white matter has also been reported from one meta-analysis of DTI studies in schizophrenia (Ellison-Wright and Bullmore, 2009), while a recent meta-analysis noted more widespread abnormalities of white matter, along with significant associations with age, duration of illness, and gender (Vitolo et al., 2017).

Recently, studies have also used dMRI to investigate short-ranged association tracts, also referred to as “U”-shaped tracts (or “U”-fibres), which reside just below the cortical surface and comprises part of the superficial white matter together with intracortical axons (d’Albis et al., 2018, Sarnat et al., 2018, Sundaram et al., 2008) in neurological and psychiatric disorders. Functionally, the “U”-fibres has been proposed to play an important role in cognitive function (Catani et al., 2012). Structurally, these tracts follow a “U”-shaped trajectory, coursing underneath the sulcus and connecting nearby gyri. Developmentally, “U”-fibres are some of the last to reach full maturation, often developing late into adulthood, with a thinner myelin sheath relative to the brain’s deep white matter (Phillips et al., 2016). Consequently, these tracts offer less protection and may be vulnerable to aberrations during development (Phillips et al., 2016). In particular, if a disorder involves generalized reduction in myelin content, then “U”-fibres may be one of the earliest to be affected, given their sparse myelination with one oligodendrocyte wrapping many “U”-fibres axon segments (Butt and Berry, 2000, Haroutunian et al., 2014). One of the challenges of examining the “U”-fibres tracts arise from the morphological differences between individuals (Thompson et al., 1996). As the tracts follow closely with the gyrification of an individual, morphological differences across individuals can introduce varying spatial arrangements of the “U”-fibres, complicating the ability to identify corresponding tracts across individuals. Previous studies have used clustering methods to first create a template of the most common “U”-fibres (e.g. those that are present in the majority of the study sample) from a sample of healthy individuals (Guevara et al., 2017, Zhang et al., 2018, Guevara et al., 2012). The created template can then be applied to identify the same “U”-fibres in other individuals, enabling the evaluation of similar short-ranged tracts.

While there has been an extensive number of studies investigating schizophrenia and associated changes using DTI, in particular the major white matter pathways, assessment of the “U”-fibres is still in its early days. Past studies involving “U”-fibres in schizophrenia have primarily examined such tracts in its entirety (e.g. average measure for a “U”-fibre), without assessing localized, along-tract changes. In this work, we utilized a template of “U”-shaped tracts we previously created (Kai and Khan, 2022) to identify and examine tracts present in the majority of patients with first-episode schizophrenia (FES) with a focus on those localized to the brain’s frontal lobe. We assessed differences in tract density, changes both along the length and in the entirety of the identified “U”-fibre tract, evaluated relationships with clinical symptoms, and identified associated networks with aberrant “U”-fibres. Through assessments along the tract length, local abnormalities that may contribute to presentation of clinical symptoms may be identified. Due to the vulnerability of “U”-fibres from the late maturation, we hypothesized that untreated patients with FES would exhibit compromised frontal “U”-fibres in the form of reduced integrity that are associated with symptom severity at the time of first presentation. Such aberrations may be more sensitive to localized changes, providing key insights into how the “U”-fibres are affected along its trajectory in FES. We also expected the affected “U”-fibres to be restricted to key functional networks relevant for cognitive control function (see (Menon and D’Esposito, 2022)) at such an early stage of psychosis, rather than affecting all of the frontal lobe. Finally, given the mounting evidence supporting a role for oligodendrocyte dysfunction in schizophrenia (see (Fessel, 2022)), we expected the “U”-fibre aberration to demonstrate changes to diffusivity measures related to tissue microstructure.

## 2 Materials and methods

All participants provided written, informed consent according to the guidelines provided by the Human Research Ethics Board for Health Sciences at Western University, London, Ontario.

### 2.1 Participants

Untreated patients with FES (n = 53; 44M/9F, ages 16–39) were recruited from referrals received by the Prevention and Early Intervention Psychosis Program (PEPP) at the London Health Sciences Center (LHSC) with an inclusion criteria of lifetime antipsychotic exposure of less than 14 days. Participants with suspected drug-induced psychosis were excluded from the study. FES participants were diagnosed with schizophrenia according to the DSM-5 criteria, using the consensus procedure described by Leckman and the Structured Clinical Interview for DSM-5 to confirm diagnosis 6 months after the first presentation (Alonso-Sánchez et al., 2022). We used a consecutive referral strategy for patient recruitment whereby all patients referred to the only first episode clinic in the catchment area between April 2017 and June 2019 were approached, if deemed to have the capacity to consent for the study by the clinicians. Healthy controls (n = 31; 19 M/12F, ages 16–29) were recruited through posters and word-of-mouth advertising, with no personal or family history of mental illness or psychotic disorders, and no current use of medications, as well as the same criteria as patients (e.g. no known neurological disorders). The healthy controls were matched for age and parental socio-economic status with the patient group. All participants were screened to exclude significant head injuries, major medical illness, or MRI contraindications. Table 1 describes the demographics and clinical characteristics for study participants. Participants for this study underwent a clinical assessment and imaging on the same day. Part of this sample has been reported in our prior studies (Alonso-Sánchez et al., 2022, Dempster et al., 2020, Limongi et al., 2020).

**Table 1:** Demographic and clinical characteristics of healthy controls and patients with first-episode schizophrenia.

**Variable**	**Controls (n = 31)**	**Patients (n = 53)**	**Controls vs Patients**
	**Demographic**		
Sex (M/F)	19/12	44/9	χ^2^ = 2.47, p = 0.12
Age [Mean (SD)]	21.55 (3.50)	23.06 (4.76)	t = −1.80, p = 0.077

	**Clinical**		
DUP [Mean (SD)]	N/A	8.20 (14.70)	
Antipsychotic Defined Daily Dose (cumulative*)	N/A	1.99 (3.10)	
SOFAS [Mean (SD)]	83.11 (4.20)	39.22 (12.19)	t = 16.4, p < 0.001
PANSS-8 Total [Mean (SD)]	8.0 (0.0)	26.39 (7.12)	t = −13.0, p < 0.001
PANSS-8 Positive [Mean (SD)]	3.0 (0.0)	11.81 (4.10)	t = −14.1, p < 0.001
PANSS-8 Negative [Mean (SD)]	3.0 (0.0)	7.36 (4.78)	t = −5.50, p < 0.001
CGI-S [Mean (SD)]	1.0 (0.0)	5.22 (1.01)	t = −20.6, p < 0.001

	**Cognitive**		
Trail Making Time (s) [Mean (SD)]	56.43 (15.23)	77.73 (34.82)	t = −2.69, p = 0.013
Trail Making Errors [Mean (SD)]	0.48 (0.85)	0.61 (1.1)	t = −0.47, p = 0.65
Category Fluency	23.74 (7.65)	17.46 (4.33)	t = 3.48, p = 0.0017

*p-values for differences between groups were calculated using chi-square for categorical variables and independent t-tests for continuous variables*.

SD: standard deviation; DUP: Duration of Untreated Psychosis (in months); SOFAS: Social and Occupational Functioning Assessment Scale; PANSS-8: Positive and Negative Syndrome Scale − 8 Item Scale; PANSS-8 Positive: PANSS-8 total score for positive symptoms; PANSS-8 Negative: PANSS-8 total score for negative symptoms; CGI-S: Clinical Global Impressions Scale - Severity *total lifetime dose exposure, calculated as Defined Daily Dose × days of exposure.

### 2.2 Clinical measures

A clinical assessment was performed by a research psychiatrist (for patients) or trained rater (for healthy controls) using the clinical battery that assessed for patient symptom severity, substance use and to confirm healthy controls had no history of psychotic illness or neurological disorder. Symptoms of psychosis were assessed using the Positive and Negative Syndrome Scale - 8 Items (PANSS-8; (Lin et al., 2018)). The abbreviated version of the assessment has been shown to be highly consistent and correlated with the full PANSS assessment consisting of 30 items (Lin et al., 2018). Items within the PANSS-8 are scored from 1 (absent) to 7 (extreme) to evaluate both positive (P1 - delusions, P2 - conceptual disorganization, P3 - hallucinations) and negative (N1 - blunted affect, N4 - passive social withdrawal, N6 - lack of spontaneity and flow of conversation) symptoms. Additionally, the severity of the illness was assessed using a subscale of the Clinical Global Impression Scale (CGI-S) (Guy, 1976). The subscale is scored between 1 (normal, not ill) to 7 (most severely ill). Furthermore, the Social and Occupational Functioning Assessment Scale (SOFAS) was conducted at the time of first presentation (Morosini et al., 2000) independent of symptom severity. SOFAS is a single-item scale scored between 1 (persistent inability to maintain minimum functioning without external support) to 100 (superior functioning in a wide range of activities). Lastly, the duration of untreated psychosis (DUP) and approximate age of onset was recorded.

### 2.3 Cognitive measures

Cognitive tests were also performed to assess different cognitive abilities for each study participant. Trail Making Test (TMT-B) was conducted, testing for the participants ability for planning, self-control, and attention. In this task, participants are asked to draw a line following a number-to-letter pattern (e.g. 1 to A to 2 to B, etc.), recording the time to completion and number of errors made during the uninterrupted task. In addition, a category fluency test was performed, testing for semantic knowledge, retrieval ability, and executive function. For this test, study participants were asked to list as many items as they could think of within a category (animals) in 60 s. The number of items listed were recorded and corrected for any repeat or non-categorical items.

### 2.4 Imaging

Imaging was performed for all study participants on a 7-Tesla (7 T) Siemens Magnetom head-only MRI system at Robarts Research Institute in London, Canada. Anatomical data was collected using a MP2RAGE sequence (Marques et al., 2010) with the following scanning parameters: repetition time/echo time (TR/TE) = 6000/2.83 ms; inversion time 1 (TI_1_) = 80 ms; inversion time 2 (TI_2_) = 2700 ms; 0.75 mm isotropic resolution; field of view (FOV) = 156 mm × 208 mm × 144 mm. Diffusion data was acquired twice with opposite phase encoding directions using the following scanning parameters: TR/TE = 5100/50.2 ms; 2.00 mm isotropic resolution; FOV = 208 mm × 208 mm × 144 mm; b-value = 1000 s/mm^2^ (64 directions) with 2b-value = 0 s/mm^2^ (non-diffusion weighted) acquisitions; multiband acceleration factor of 2.

### 2.5 Processing

**Fig. 1:**
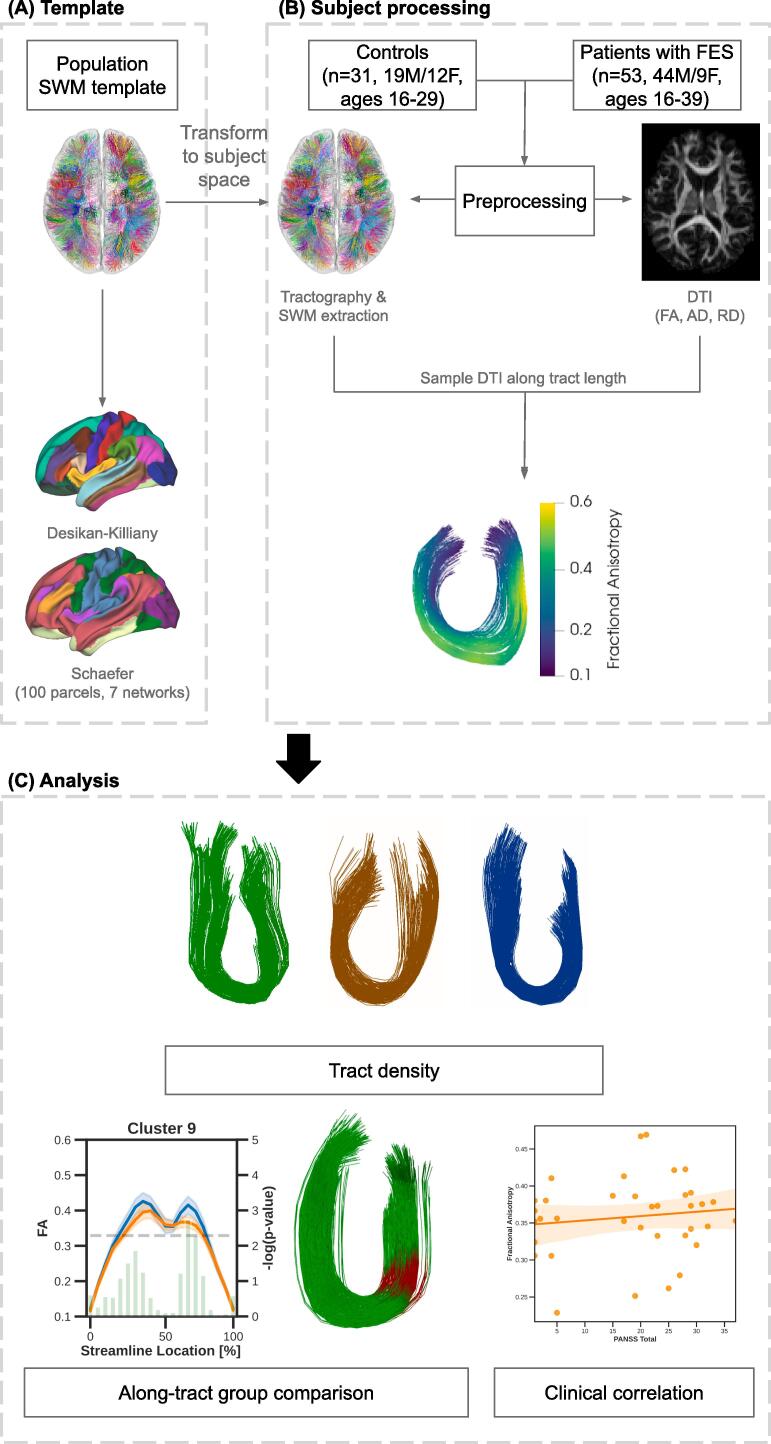
Overview of processing and analysis workflow. (A) A population SWM template was registered to each subject’s native space to aid identification of corresponding tracts across subjects.“U”-shaped tracts constrained to the frontal lobe was mapped to anatomical (Desikan-Killiany) and functional (Schaefer - 100 parcels, 7 networks) parcellations. (B) Data collected from healthy controls and patients with FES were preprocessed before performing tractography to identify frontal lobe SWM and deriving quantitative maps from DTI. Derived metrics were mapped along the length of the identified frontal lobe SWM. (C) Analysis was performed on clusters that were present in 70% of each group, evaluating for tract density and quantitative differences. Correlations between clinical measures and derived metrics was performed to identify relationships in patients.

Image data processing was performed in containerized computing environments on high performance compute clusters. The following subsections detail the processing steps performed. An overview of the general workflow performed is shown in Fig. 1. Briefly, data collected using an MRI was used to identify and assess “U”-fibres of the frontal lobe using tractography. Analysis included assessment of both tract density and along-tract differences from DTI derived measures, as well as evaluation of the relationship of clinical measures and DTI derived measures. Furthermore, affected tracts were mapped to the associated anatomical regions and functional networks.

#### 2.5.1 Anatomical T1-weighted

Data was preprocessed by first applying a correction to unwarp distorted anatomical volumes due to gradient field inhomogeneities (gradient non-linearity correction). This was performed using a modified version of publicly available code^2^ to work with a proprietary scanner-specific file. Following gradient non-linearity correction, fMRIprep (v1.5.4) was applied to each subject’s anatomical data (Esteban et al., 2019, Esteban et al., 2020) for further preprocessing. The preprocessing includes the correction for intensity non-uniformity, and skull stripping. As part of the fMRIprep pipeline, cortical segmentations were derived with FreeSurfer (Dale et al., 1999).

#### 2.5.2 Diffusion MRI (dMRI)

First, distortion correction due to gradient field inhomogeneities was performed using a modified version of publicly available code with a proprietary scanner-specific file, similar to the anatomical preprocessing step. Then, dMRI data was preprocessed with prepdwi (Khan et al., 2021), a pipeline developed in-house consisting of principal component analysis based denoising (Veraart et al., 2016a, Veraart et al., 2016b) and minimization of Gibbs ringing effects (Kellner et al., 2016) followed by correcting for distortions induced by susceptibility, eddy currents, and subject motion through application of FSL’s topup (Andersson et al., 2003, Smith et al., 2004) and eddy (Andersson and Sotiropoulos, 2016). The preprocessed dMRI data was rigidly registered with the subject's anatomical data using Niftyreg^3^ as part of the in-house preprocessing pipeline. Using Mrtrix3, diffusion tensors were estimated from the preprocessed dMRI data and fractional anisotropy (FA), radial diffusivity (RD), axial diffusivity (AD), and mean diffusivity (MD) maps were derived using an iteratively reweighted linear least squares estimator (Veraart et al., 2013).

#### 2.5.3 Tractography

Following dMRI preprocessing, Mrtrix3 (Tournier et al., 2019) was used to further process the data for tractography. First, individual subject response functions were estimated using the Dhollander algorithm (Dhollander et al., 2016) and group average response functions were created from healthy controls. Individual subject fibre orientation distribution (FOD) maps were computed from the average response functions using the multi-shell, multi-tissue algorithm (Jeurissen et al., 2014) while excluding the GM response function for single-shell data, enabling comparison of corresponding tracts between individuals (Raffelt et al., 2012). By excluding the GM response function, the multi-shell, multi-tissue algorithm can still be used for single-shell data in order to suppress signal from the cerebrospinal fluid. A population template previously created from unrelated subjects, including FOD maps and labeled “U”-fibre tractography (Kai and Khan, 2022) was used to aid identification of “U”-shaped tracts. To do so, individual subject FOD maps were first registered to the population FOD template using a multi-resolution pyramid structure to determine transformations between the template and subject spaces. Using the transformation from the template to the subject’s native space, the population template was transformed to each subject’s native space to identify and establish correspondence of tracts between subjects.

Whole-brain probabilistic tractography was performed for each subject using a tensor-based algorithm (Jones, 2008) identifying streamlines of all tracts, including “U”-fibres and other major tracts, again using the Mrtrix3 software suite (Tournier et al., 2019). Seeding of the tractography was performed at random within the brain until a target of 10,000,000 streamlines had been selected. Following creation of the tractogram from the tensor-based algorithm, streamlines were filtered via spherical-deconvolution informed filtering (SIFT) to match WM FOD amplitudes until a target of 1,000,000 streamlines remained for each subject (Smith et al., 2013).

Short-ranged, "U"-shaped streamlines were identified from whole-brain tractography using established parameters for determining a “U”-shaped trajectory (streamline length between 20 mm and 80 mm and the distance between streamline endpoints was approximately 1/3 of the streamline length) (O’Halloran et al., 2017). Streamlines with “U”-shaped trajectories were clustered into tracts through label propagation from the previously labeled template with streamlines assigned to the cluster of the most similar template tract. Additionally, streamlines were mapped to the nearest Freesurfer-based lobar parcellation within a 4 mm radius of the terminal ends. For tracts with streamlines connecting more than two nodes, tracts were assigned to the two nodes where the majority of the streamlines terminated. From here, “U”-fibres residing in the frontal lobe were identified for further analysis. Moreover, quantitative values derived from DTI were mapped to 20 equidistant samples on each streamline of frontal lobe “U”-shaped tracts and averaged to create a mean along-tract measurement for tractometry analysis. Furthermore, a single mean quantitative measurement was also computed for each identified tract by averaging the sampled measurements in associated streamlines to evaluate overall tract changes.

#### 2.5.4 Surface and parcellation mapping

Each template “U”-shaped tract was mapped to a population average symmetric brain surface mesh with approximately 32k vertices (named fs_LR32k). First, the centroid of each terminal end was identified and the Euclidean distance to the surface vertices was computed. A labeled surface map was created by assigning the label of the nearest tract to each vertex. To identify associated brain regions, anatomical and functional atlases were used. Specifically, the Desikan-Killiany (Desikan et al., 2006) and the Schaefer atlases ((Schaefer et al., 2018); 100 parcellations, 7 networks) were used to determine the associated anatomical parcels and functional networks respectively for each identified “U”-shaped tract.

### 2.6 Analysis

For analysis, identified frontal lobe tracts were discarded if fewer than 5 streamlines were present. Furthermore, individual variability may reduce the capability of identifying corresponding “U”-fibres across study participants from the previously created template. As such, tracts were only considered for analysis if they were determined to be present in at least 70% of the healthy control and patient groups respectively.

#### 2.6.1 Tract density

Individual streamlines were extracted and summed to determine the tract density of identified tracts meeting analysis criteria. Tract densities were regressed to control for age and sex covariates and a Bartlett test was performed to determine whether the variance of the two groups were equal, followed by a two-sided, Welch’s *t*-test (Welch, 1947) to test for differences in density of individual tracts between groups (due to unequal variance between healthy controls and patients). False discovery rate correction was performed with the Benjamini-Hochberg procedure for multiple comparisons (Benjamini and Hochberg, 1995). Furthermore, a Welch’s *t-*test was performed to compare the total density comprising frontal lobe “U”-fibres.

#### 2.6.2 Microstructural changes

Measures of FA, RD, AD, and MD sampled equidistantly along the length of tract were regressed to control for age and sex covariates prior to analysis and a Welch’s *t*-test was performed to compare measured samples between groups. To account for the numerous comparisons carried out between samples, as well as across tracts, a permutation-based multiple comparison correction approach with 10,000 repetitions was applied to adjust the significance threshold (Nichols and Holmes, 2002), similar to (Colby et al., 2012, Wasserthal et al., 2020). Using this procedure, an adjusted threshold of p < 0.005 was determined to be significant for along-tract measures in this study.

#### 2.6.3 Relationships with clinical and cognitive measures

An average quantitative measure (i.e., mean FA, RD, AD, MD) was computed from the segments identified with differences along the length of the tract between the two groups and the relationships with included clinical measures of PANSS-8, SOFAS, DUP, CGI-S, and age of onset were evaluated using Pearson’s correlation in the patient cohort. Additionally, relationships with cognitive measures of TMT-B completion time, error rate, and category fluency score were also evaluated using Pearson’s correlations in the patient cohort. As with tract density, false discovery rate correction was performed using the Benjamini-Hochberg procedure to correct for multiple comparisons.

## 3 Results

Comparing demographic and clinical measures between healthy controls and patients with FES, no statistically significant differences were observed for sex or age. However, within the patient group, males were over-represented (χ^2^ = 15.364, p < 0.001). Patients were also observed to demonstrate a statistically significant difference in SOFAS scores (t = 16.4, p < 0.001), PANSS-8 Total (t = −13.0, p < 0.001), PANSS-8 Positive (t = −14.1, p < 0.001), PANSS-8 Negative (t = 5.50, p < 0.001), and CGI-S (t = −20.6, p < 0.001) scores. Differences were also observed for cognitive measures of trail making completion time (t = −2.69, p = < 0.05) and category fluency (t = 3.48, p < 0.05).

### 3.1 Tract density of frontal lobe “U”-fibres

**Fig. 2:**
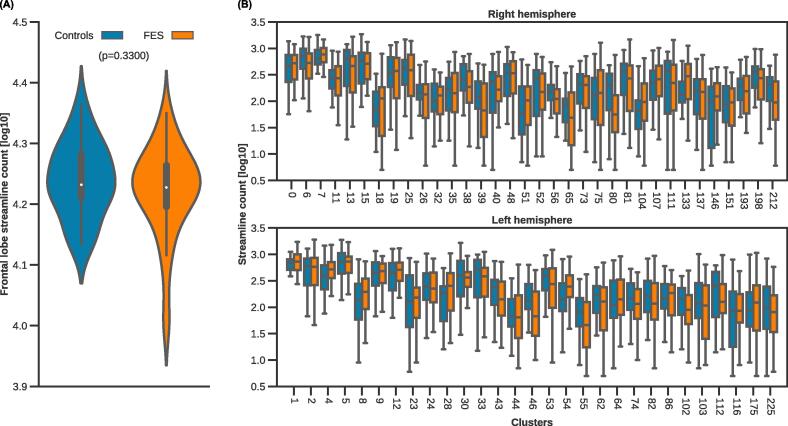
(A) Log-transformed distribution of frontal lobe SWM streamline counts from tracts present in 70% of healthy controls and patients with FES. No differences were observed. (B) Log-transformed distributions of individual frontal lobe “U”-shaped tracts in the right (top) and left (bottom) hemispheres for controls and patients. Following correction for false discovery (via Benjamini-Hochberg), no differences were observed.

After applying the previously mentioned constraints (“U”-shaped trajectory residing within the frontal lobe; see section 2.4.3), 63 out of 122 frontal lobe “U”-shaped tracts across both left and right hemispheres were retained. Average streamline counts from these tracts throughout the frontal lobe were assessed between controls and patients with FES. Performing a Welch’s *t*-test yielded no significant differences between the two groups following correction for multiple comparisons, indicating that in schizophrenia, the density (in this case the overall volume inferred from diffusion-based streamline count) was unaffected in frontal “U”-fibres. Fig. 2 exhibits the assessed tract densities described (log-transformed for visualization).

### 3.2 Affected “U”-fibres and associated structural and functional parcellations

**Fig. 3:**
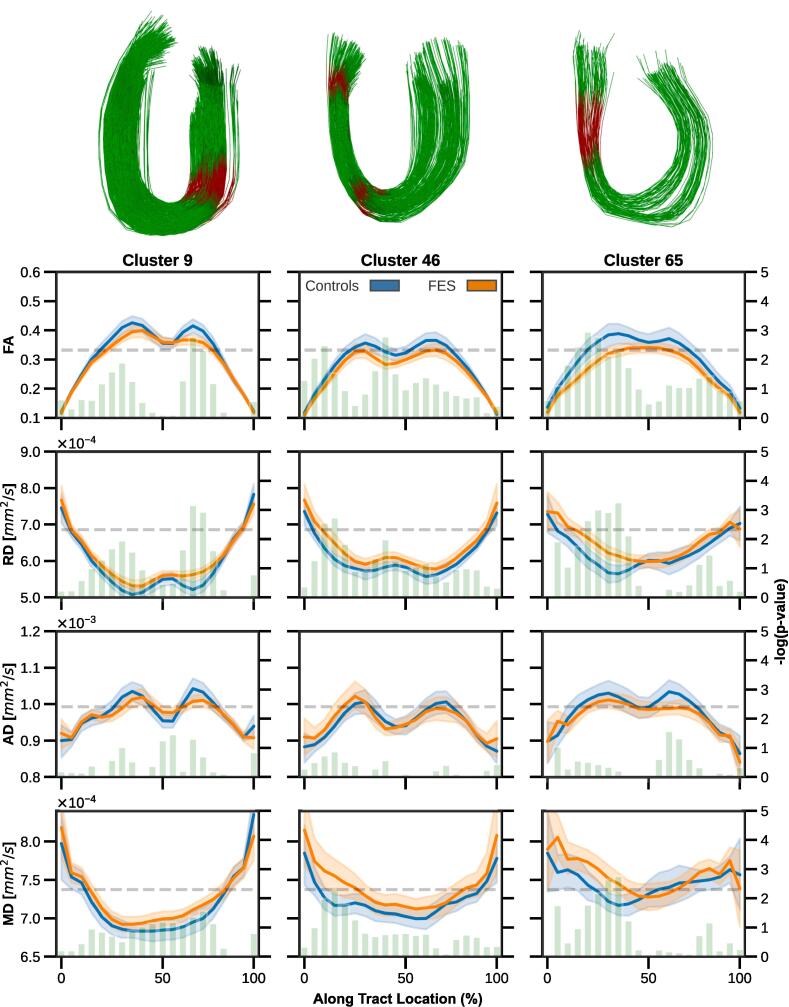
DTI-derived measures (top: FA, middle-top: RD, middle-bottom: AD, bottom: MD) mapped along frontal lobe tracts of controls (blue) and patients (orange). Segments with observed differences between groups are highlighted (in red). 3 tracts were identified (2 in left hemisphere, 1 in right hemisphere) with increased FA and decreased RD.

From the frontal lobe “U”-fibres that met the defined analysis criteria, tractometry analysis identified 3 tracts (2 in the left hemisphere, 1 in the right hemisphere) with significant along-tract DTI derived differences after performing a permutation-based multiple comparisons correction. All 3 tracts (Fig. 3) demonstrated a significant decrease in FA (p < 0.005), together with a significant increase in RD along similar segments (p < 0.005) in patients with FES, indicating localized deficits in regards to tissue microstructure. More details related to these affected segments can be found in the Supplementary Material. The affected segments were detected adjacent to the midpoint of the tract, nearest to the sulci. A slight increase in MD was also observed along similar segments in patients with FES, however these observations were not determined to be statistically significant. No significant differences in AD were identified for any analyzed tracts, indicating a relative preservation of axonal integrity at this early stage of psychosis.

**Fig. 4:**
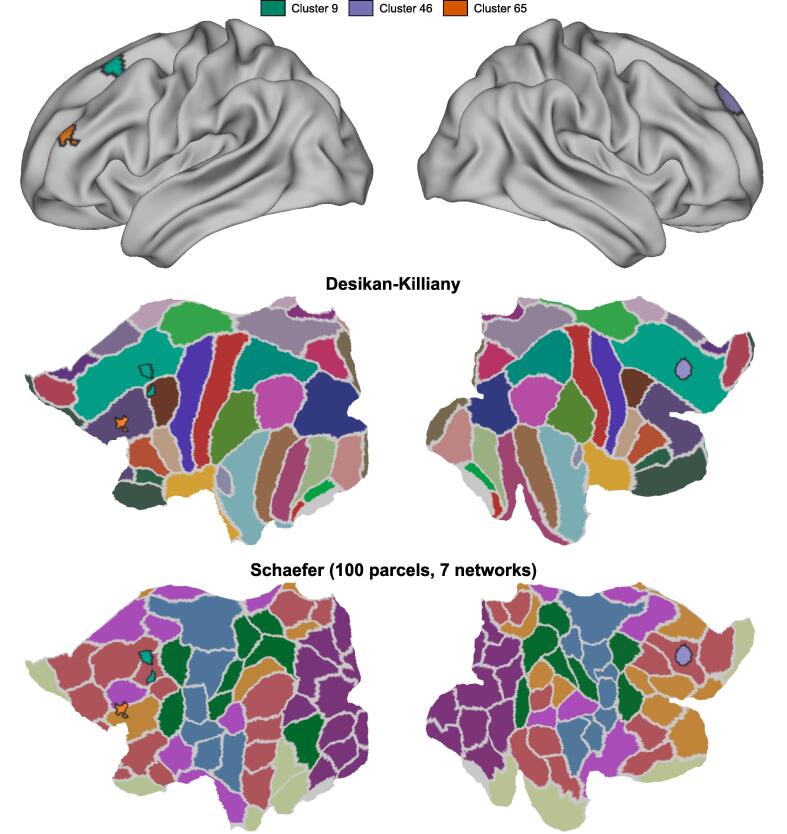
Frontal lobe SWM mapped to the fs LR32k white matter surface (top). Identified frontal lobes with decreased FA and increased RD are outlined in black on flat maps of anatomical (middle; Desikan-Killiany) and functional (bottom; Schaeffer) parcellations. The “U”-shaped tracts were associated with default mode, frontoparietal, and ventral attention functional networks.

Each affected tract was also mapped (Fig. 4) to both anatomical parcellations (from the Desikan-Killiany atlas) and functional network parcellations (from the Schaefer atlas containing 7 networks, 100 parcels). Tract 9, one of the identified affected frontal lobe tracts in the left hemisphere, was found to connect the superior frontal and caudal middle frontal brain parcels and associated with the default mode (DMN) functional network. The other identified frontal lobe connection of the left hemisphere, Tract 46, resided entirely within the caudal middle frontal brain parcel, interconnecting with the frontoparietal (FPN) and ventral attention (VAN) functional networks. Lastly, Tract 65 was identified to reside within the superior frontal parcel of the right hemisphere, and also associated with the DMN.

### 3.3 Correlations between DTI and clinical measures

Segments along the tract where differences were observed for each tract were averaged to obtain a single measure and correlated with clinical measures (e.g. PANSS-8, CGI-S, SOFAS, DUP, approximate age of onset). Table 2 outlines the correlations of measures from affected tract segments and the clinical measures. No significant correlations were identified between DTI measures (FA, RD, MD) and clinical measures. Correlations were not performed against MD for tract 9 or AD as there were no significant segments identified.

**Table 2:** Correlations between DTI-derived measures (from aberrant segments along-tract) and clinical characteristics in patients with first-episode schizophrenia. MD of tract 9 and AD not included (no identified aberrant segments).

**Variable**	**Tract 9**								
	**FA**			**RD**			**MD**		
	**r**	**p**	**p**_**corrected**_	**r**	**p**	**p**_**corrected**_	**r**	**p**	**p**_**corrected**_
Age	0.234	0.307	0.911	−0.327	0.148	0.911			
CGI-S	−0.141	0.543	0.911	0.237	0.302	0.949			
DUP	0.185	0.422	0.911	−0.190	0.409	0.911			
SOFAS	0.304	0.180	0.844	−0.316	0.163	0.845			
PANSS-8 Total	−0.376	0.093	0.911	0.318	0.160	0.911			
PANSS-8 Positive	−0.316	0.163	0.911	0.332	0.142	0.869			
PANSS-8 Negative	−0.173	0.453	0.911	0.082	0.723	0.911			

	**Tract 46**								

	**r**	**p**	**p_corrected_**	**r**	**p**	**p_corrected_**	**r**	**p**	**p_corrected_**

Age	0.151	0.562	0.845	0.187	0.473	0.911	0.466	0.0595	0.845
CGI-S	−0.302	0.239	0.845	0.353	0.164	0.845	0.210	0.420	0.845
DUP	−0.258	0.317	0.911	−0.087	0.739	0.845	−0.081	0.901	0.911
SOFAS	0.056	0.831	0.949	−0.298	0.245	0.845	−0.252	0.328	0.845
PANSS-8 Total	−0.113	0.666	0.911	−0.008	0.973	0.911	−0.172	0.509	0.911
PANSS-8 Positive	−0.247	0.339	0.845	0.228	0.379	0.911	−0.046	0.861	0.911
PANSS-8 Negative	0.226	0.302	0.911	−0.371	0.142	0.845	−0.238	0.358	0.845

	**Tract 65**								

	**r**	**p**	**p_corrected_**	**r**	**p**	**p_corrected_**	**r**	**p**	**p_corrected_**

Age	−0.203	0.419	0.911	−0.103	0.683	0.962	−0.163	0.518	0.911
CGI-S	0.421	0.0820	0.690	−0.426	0.0781	0.690	−0.275	0.269	0.690
DUP	0.081	0.749	0.949	0.001	0.996	0.962	0.032	0.901	0.949
SOFAS	−0.276	0.268	0.690	−0.053	0.835	0.690	−0.273	0.274	0.845
PANSS-8 Total	0.005	0.984	0.962	4.25E-06	0.999	0.922	0.105	0.679	0.911
PANSS-8 Positive	0.119	0.664	0.921	−0.032	0.899	0.962	0.107	0.674	0.911
PANSS-8 Negative	−0.157	0.534	0.911	0.051	0.840	0.962	0.018	0.945	0.911

r: Pearson's correlation coefficient; p: Uncorrected p-value; p_corrected_: Corrected p-value; CGI-S: Clinical Global Impressions Scale - Severity; DUP: Duration of Untreated Psychosis (in months); SOFAS: Social and Occupational Functioning Assessment Scale; PANSS-8: Positive and Negative Syndrome Scale - 8 Item Scale; PANSS-8 Positive: PANSS-8 total score for positive symptoms; PANSS-8 Negative: PANSS-8 total score for negative symptoms;

### 3.4 Correlations between DTI and cognitive measures

Similar to section 3.3, correlations were performed between an average of the aberrant segments and cognitive measures (e.g. trail making completion time, trail making errors, category fluency). Correlations between measures from affected tract segments and cognitive measures can be found in Supplementary Table S1. A significant correlation was identified between the number of errors made during the trail making task and FA from tract 46 prior to multiple comparisons correction. No other significant correlations were identified both prior to and after multiple comparisons correction.

## 4 Discussion

### 4.1 Frontal lobe “U”-fibre DTI abnormalities

As part of our study focused on frontal lobe “U”-fibres, we examined changes of DTI derived metrics along the tract trajectory in patients with FES, which enabled detection of a reduction in FA and increased RD (Fig. 3). In 3 other studies of “U”-fibres using DTI, a similar reduction, but for tract-averaged FA was observed in patients with schizophrenia (Ji et al., 2019, Nazeri et al., 2013, Phillips et al., 2011). While there are differences between the current study and the studies mentioned (e.g. examination localized to the frontal lobe, assessment of patients with FES), our findings are in agreement with previous studies. Tract density differences were also evaluated, but no differences between patients and healthy controls were identified. The combination of reduced FA (indicative of less restricted diffusion), and increased RD (implying more diffusion in secondary and tertiary directions to axons), but preserved apparent density (streamline count) may relate to affected tissue microstructure in the frontal lobe “U”-fibres of patients with FES. Localized aberrations in myelin content may be a potential explanation, but this inference is tentative given the lack of direct correspondence between DTI measures and myelin (MacKay and Laule, 2016). While our analysis is unable to specifically identify or determine the timing of the phenomena that is occurring with respect to the onset of psychosis in the three affected “U”-shaped tracts in the frontal lobe, given the nature of our sample, we can conclude that this effect precedes prolonged exposure to antipsychotics and other secondary physical and social effects of chronic illness.

The development of the short-ranged connections have also been observed to lag behind typical maturation in patients with schizophrenia relative to healthy individuals (Ouyang et al., 2017). Furthermore, a lack of association with attention, working memory, and processing speed has been noted in “U”-fibres throughout the brain, which are thought to be associated with higher-order cognitive functions (Ouyang et al., 2017, Yoshino et al., 2020), with schizophrenia when compared against healthy controls (Nazeri et al., 2013). In the present study, a modest association was found between FA of a frontal lobe “U”-fibre and the number of errors made during TMT-B (which tests for planning, self-control and attention), but this did not withstand multiple comparisons correction. The degree of noted abnormalities (increased FA, reduced RD) in the present study, examined only in frontal “U”-fibres, may be insufficient to influence higher-order cognitive functions or be an invariant, albeit modest change irrespective of cognitive deficits in patients with schizophrenia. Our observations, in addition to previous studies of “U”-fibres in schizophrenia, may provide support for the hypothesis of developmental abnormalities due to the late maturation of “U”-fibres (Phillips et al., 2016).

### 4.2 Localized aberrations in frontal lobe “U”-fibres

Irregularities observed along affected may be a contributing factor to abnormal interactions between brain regions, one of the hypotheses underpinning symptoms of psychosis (Fornito et al., 2012, Stephan et al., 2009, Wernicke, 1906). Studies of functional networks, associated with brain activity and identified by statistical correlation of neurophysiological time series data, have corroborated abnormal interactions between brain regions this hypothesis identifying differences in networks of patients with schizophrenia when compared against healthy controls (e.g. (Jiang et al., 2019, Kraguljac et al., 2016, Weinberger et al., 1986)**)**. Widespread regional DTI abnormalities have also been previously observed in studies of psychosis (Kelly et al., 2018, Vitolo et al., 2017). As part of the current study, we mapped the identified frontal lobe “U”-fibres to both the nearest anatomic parcels (Desikan-Killiany atlas) and functional networks (Schaefer atlas - 100 parcels, 7 networks).

In this study, 3 frontal lobe “U”-shaped tracts were identified with localized abnormalities related to tissue microstructure, with 2 tracts residing in the left hemisphere: (1) Tract 9 - associated with the superior frontal and caudal middle frontal parcels and functionally associated with DMN and (2) Tract 46 - associated with the caudal middle frontal parcel and functionally associated with both the FPN and VAN. In the opposite hemisphere, (3) Tract 65 was observed to be associated with the superior frontal parcel and functionally associated with DMN. Moreover, the associated functional networks of the affected tracts have been previously shown to have altered activity in schizophrenia (Hummer et al., 2020, Mingoia et al., 2012, Phillips et al., 2011).

Previous post-mortem studies have also demonstrated a significant reduction of oligodendrocytes, responsible for the formation of myelin, in patients with schizophrenia (Byne et al., 2008, Hof et al., 2003, Mighdoll et al., 2015, Stark et al., 2004). Specifically, a decrease in the number of oligodendrocytes, as well as altered spatial arrangement was observed in the superior frontal gyrus in patients with schizophrenia (Hof et al., 2003). Oligodendrocyte abnormalities and ultimately the myelin anomalies may be related to oxidative stress, which have a downstream effect on oligodendrocyte precursor cells (Maas et al., 2017). Increased oxidative stress can lead to the inability to produce myelin due to apoptosis of oligodendrocyte precursor cells that are transitioning to oligodendrocytes and has been suggested to be a likely cause of lack of myelination in schizophrenia (Maas et al., 2017). The late maturing “U”-fibres is likely affected by the consequences of increased oxidative stress during its development and may be related to the observed anomalies in affected frontal lobe “U”-fibres.

Another circumstance of the observed DTI characteristics may be due to cell swelling, which has been observed in the prefrontal brain region of patients with schizophrenia (Uranova et al., 2011). Such phenomena has been associated with increased radial diffusivity (Aung et al., 2013), which was suggested in the observed DTI anomalies that were identified along the tract length (see section 3.2). Other possibilities include a change in membrane permeability or due to a change in axon diameter. Aberrations, such as those observed in the present study, may be linked with abnormal interactions between the different brain regions and lead to a deficit of higher order cognitive functions.

### 4.3 Imaging and non-imaging confounds

The present study recruited participants that were age and socio-economically matched with no history of psychotic disorders or known neurological disorders and no current use of medications. However, other confounding factors may have contributed to noted observations such as use of recreational drugs (e.g. cannabis), smoking, and drinking. Moreover, environmental risk factors (e.g. birthing complications, parental age) and genetic factors that have been associated with schizophrenia were not considered in the present study and may have had an influence on observations. Previous studies have provided evidence of substance use inducing psychosis, as well as association of schizophrenia with other risk factors (Mäki et al., 2005, Stilo and Murray, 2019).

In addition to non-imaging confounds, models such as DTI, which is commonly applied to investigate changes of the microstructural environment also suffer from limitations. Measures derived from DTI can be confounded by multiple tract orientations within a voxel (e.g. cause a reduction in FA), as well as be influenced by partial volume effects (e.g. more than a single tissue type within a voxel). The latter may affect “U”-fibre studies and associated along-tract measures as these tracts are found near the cortical surface with trajectories that follow gyral patterns. Future studies of “U”-fibres in FES may benefit from using stronger diffusion gradients and multiple diffusion shells to perform higher order modeling (e.g. neurite orientation dispersion and density imaging (Zhang et al., 2012)), potentially allowing for probing of different tissue compartments to gain further insight on changes to “U”-fibres.

### 4.4 Limitations

This study of frontal lobe “U”-fibres in patients with FES is not without limitations. One challenge was the identification of corresponding “U”-shaped tracts while accounting for variations for morphological differences across individuals. To address this, we used a population-based template, examining frontal lobe tracts with similar trajectories as those from the template. An arbitrary threshold of 70% was used to identify frontal lobe “U”-shaped tracts as possible in the majority of both healthy control and patient groups. Consequently, this resulted in evaluation of just over half of all identified frontal lobe tracts, leaving out other potentially important “U”-fibres both in the frontal lobe and in other regions of the brain. In previous psychosis studies of “U”-fibres, similar approaches have been used, examining tracts present in either all or the majority of the study participants (Guevara et al., 2017, Ji et al., 2019). As knowledge of “U”-fibres is gained, future work may be able to identify corresponding “U”-fibres across individuals irrespective of morphological differences, relying on functional characterization of the “U”-shaped tracts. One such promising method was recently presented, using surface-based tractography techniques to identify the “U”-shaped tracts (Shastin et al., 2022).

It has been noted that schizophrenia, which typically develops in early adulthood, tends to have a higher clinical prevalence in men than in women (Jauhar et al., 2022). While there was no significant imbalance within sex distribution comparing healthy controls and FES patients (χ^2^ = 2.47, p = 0.116), males were over-represented in the sample of patients (χ^2^ = 15.36, p < 0.001) in the present study. Consequently, observed differences may be biased towards male patients. Future studies should explore potential differences in “U”-fibres between males and females in FES.

In the present study, we performed a correlation of derived DTI measures from the affected segments of the frontal lobe “U”-fibres with clinical measures, where we did not observe any significant correlations following multiple comparisons correction. In contrast, a previous study of “U”-fibres in patients with schizophrenia determined a single “U”-shaped tract connecting the postcentral and supramarginal gyri to be correlated with negative symptoms (Ji et al., 2019). Furthermore, meta-analyses have identified widespread DTI anomalies in patients with schizophrenia (Kelly et al., 2018, Vitolo et al., 2017). Our observation of a lack of symptom correlations of frontal lobe “U”-fibres with clinical measures may be attributed to integrity of “U”-fibres being an invariant feature irrespective of symptom severity in patients with FES. Another possibility is the lack of sufficient power to identify a relationship between the DTI and clinical features due to the sample size of the present study.

We also performed a correlation of derived DTI measures with cognitive measures, where we also did not observe any significant correlations following multiple comparisons correction in patients with FES. However, in a previous study of “U”-fibres in patients with schizophrenia, it had been suggested that the observed DTI differences were found in regions associated with executive functions and working memory (Ji et al., 2019). As previously noted, one possibility of the negative finding is due to a lack of sufficient power resulting in an inability to identify relationships between white matter and cognitive deficits, which have been observed in large-scale studies of major white matter tracts of patients with schizophrenia (Kelly et al., 2018).

## 5 Conclusions

We demonstrated quantitative changes that occur irrespective of symptom severity to the frontal lobe “U”-fibres in schizophrenia. The simultaneous decrease in FA and increase in RD suggests abnormalities in regards to the tissue microstructure of frontal lobe “U”-fibres, disrupting normal brain connectivity in patients with FES. Factors such as decreased oligodendrocytes (responsible for myelin formation) may contribute to the observed changes in patients with FES. Our observations from early stages of illness support the primary role for prefrontal disconnection and highlight the potential of tracking the progression of measures related to the tissue microstructure in the short-ranged “U”-shaped fibres in patients with schizophrenia to establish their utility in marking the course of illness. Future work should explore the phenomena underlying the differences in DTI measures, identify affected “U”-fibres in other brain regions, assess longitudinal changes to the “U”-fibres, and further investigate the function of “U”-fibres and its relationship to cognition.

## Data availability

The data from this study are available from the corresponding author (LP) upon reasonable request.

## CRediT authorship contribution statement

**Jason Kai:** Methodology, Formal analysis, Writing – original draft. **Michael Mackinley:** Data curation, Writing - review & editing. **Ali R. Khan:** Methodology, Resources, Writing – review & editing. **Lena Palaniyappan:** Conceptualization, Writing - review & editing.

## Declaration of Competing Interest

The authors declare the following financial interests/personal relationships which may be considered as potential competing interests: LP reports personal fees for serving as chief editor from the Canadian Medical Association Journals, speaker/consultant fee from Janssen Canada and Otsuka Canada, SPMM Course Limited, UK, Canadian Psychiatric Association; book royalties from Oxford University Press; investigator-initiated educational grants from Janssen Canada, Sunovion and Otsuka Canada outside the submitted work. Other authors have no conflicts of interest to declare.

## Appendix A Supplementary data

Supplementary data 1 Details the quantitative measures observed along the length identified aberrant tracts.

## Footnotes

2 https://github.com/kaitj/gradunwarp/.

3 https://cmictig.cs.ucl.ac.uk/wiki/index.php/NiftyReg.

## References

[b0005] Alonso-Sánchez M.F., Ford S.D., MacKinley M., Silva A., Limongi R., Palaniyappan L. (2022). Progressive changes in descriptive discourse in First Episode Schizophrenia: a longitudinal computational semantics study. Schizophrenia.

[b0010] Andersson J.L.R., Skare S., Ashburner J. (2003). How to correct susceptibility distortions in spin-echo echo-planar images: application to diffusion tensor imaging. Neuroimage.

[b0015] Andersson J.L.R., Sotiropoulos S.N. (2016). An integrated approach to correction for off-resonance effects and subject movement in diffusion MR imaging. Neuroimage.

[b0020] Aung W.Y., Mar S., Benzinger T.LS. (2013). Diffusion tensor MRI as a biomarker in axonal and myelin damage. Imaging Med..

[b0025] Basser P.J., Mattiello J., LeBihan D. (1994). MR diffusion tensor spectroscopy and imaging. Biophys. J..

[b0030] Benjamini Y., Hochberg Y. (1995). Controlling the false discovery rate: A practical and powerful approach to multiple testing. J. R. Stat. Soc. B. Methodol..

[b0035] Butt A.M., Berry M. (2000). Oligodendrocytes and the control of myelination in vivo: new insights from the rat anterior medullary velum. J. Neurosci. Res..

[b0040] Byne W., Tatusov A., Yiannoulos G., Vong G., Marcus S. (2008). Effects of mental illness and aging in two thalamic nuclei. Schizophr. Res..

[b0045] Catani M., Dell’Acqua F., Vergani F., Malik F., Hodge H., Roy P., Valabregue R., Thiebaut de Schotten M. (2012). Short frontal lobe connections of the human brain. Cortex.

[b0050] Colby J.B., Soderberg L., Lebel C., Dinov I.D., Thompson P.M., Sowell E.R. (2012). Along-tract statistics allow for enhanced tractography analysis. Neuroimage.

[b0055] d’Albis M.-A., Guevara P., Guevara M., Laidi C., Boisgontier J., Sarrazin S., Duclap D., Delorme R., Bolognani F., Czech C., Bouquet C., Ly-Le Moal M., Holiga S., Amestoy A., Scheid I., Gaman A., Leboyer M., Poupon C., Mangin J.-F., Houenou J. (2018). Local structural connectivity is associated with social cognition in autism spectrum disorder. Brain.

[b0060] Dale A.M., Fischl B., Sereno M.I. (1999). Cortical surface-based analysis. I. Segmentation and surface reconstruction. NeuroImage.

[b0065] Dempster K., Jeon P., MacKinley M., Williamson P., Théberge J., Palaniyappan L. (2020). Early treatment response in first episode psychosis: a 7-T magnetic resonance spectroscopic study of glutathione and glutamate. Mol. Psychiatry.

[b0070] Desikan R.S., Ségonne F., Fischl B., Quinn B.T., Dickerson B.C., Blacker D., Buckner R.L., Dale A.M., Maguire R.P., Hyman B.T., Albert M.S., Killiany R.J. (2006). An automated labeling system for subdividing the human cerebral cortex on MRI scans into gyral based regions of interest. Neuroimage.

[b0075] Dhollander T., Raffelt D., Connelly A. 2016. Unsupervised 3-tissue response function estimation from single-shell or multi-shell diffusion MR data without a co-registered T1 image. In: Proceedings of ISMRM Workshop on Breaking the Barriers of Diffusion MRI. Lisbon, Portugal. p 5.

[b0080] Ellison-Wright I., Bullmore E.d. (2009). Meta-analysis of diffusion tensor imaging studies in schizophrenia. Schizophr. Res..

[b0085] Esteban O., Markiewicz C.J., DuPre E., Goncalves M., Kent J.D., Ciric R., Blair R.W., Poldrack R.A., Gorgolewski K.J. 2020. fMRIPrep: a robust preprocessing pipeline for functional MRI. Zenodo. https://zenodo.org/record/3700055. 10.1038/s41592-018-0235-4 PMC6319393 30532080

[b0090] Esteban O., Markiewicz C.J., Blair R.W., Moodie C.A., Isik A.I., Erramuzpe A., Kent J.D., Goncalves M., DuPre E., Snyder M., Oya H., Ghosh S.S., Wright J., Durnez J., Poldrack R.A., Gorgolewski K.J. (2019). fMRIPrep: a robust preprocessing pipeline for functional MRI. Nat. Methods.

[b0095] Fessel J. (2022). Abnormal oligodendrocyte function in schizophrenia explains the long latent interval in some patients. Transl. Psychiatry.

[b0100] Fornito A., Zalesky A., Pantelis C., Bullmore E.T. (2012). Schizophrenia, neuroimaging and connectomics. Neuroimage.

[bib386] Guevara P., Duclap D., Poupon C., Marrakchi-Kacem L., Fillard P., Le Bihan D., Leboyer M., Houenou J., Mangin J.-F. (2012). Automatic fiber bundle segmentation in massive tractography datasets using a multi-subject bundle atlas. Neuroimage.

[b0105] Guevara M., Román C., Houenou J., Duclap D., Poupon C., Mangin J.F., Guevara P. (2017). Reproducibility of superficial white matter tracts using diffusion-weighted imaging tractography. Neuroimage.

[b0110] Guy W. (1976).

[b0115] Haroutunian V., Katsel P., Roussos P., Davis K.L., Altshuler L.L., Bartzokis G. (2014). Myelination, oligodendrocytes, and serious mental illness. Glia.

[b0120] Hof P.R., Haroutunian V., Friedrich V.L., Byne W., Buitron C., Perl D.P., Davis K.L. (2003). Loss and altered spatial distribution of oligodendrocytes in the superior frontal gyrus in schizophrenia. Biol. Psychiatry.

[b0125] Hummer T.A., Yung M.G., Goñi J., Conroy S.K., Francis M.M., Mehdiyoun N.F., Breier A. (2020). Functional network connectivity in early-stage schizophrenia. Schizophr. Res..

[b0130] Jauhar S., Johnstone M., McKenna P.J. (2022). Schizophrenia. Lancet.

[b0135] Jeurissen B., Tournier J.-D., Dhollander T., Connelly A., Sijbers J. (2014). Multi-tissue constrained spherical deconvolution for improved analysis of multi-shell diffusion MRI data. Neuroimage.

[b0140] Ji E., Guevara P., Guevara M., Grigis A., Labra N., Sarrazin S., Hamdani N., Bellivier F., Delavest M., Leboyer M., Tamouza R., Poupon C., Mangin J.-F., Houenou J. (2019). Increased and decreased superficial white matter structural connectivity in schizophrenia and bipolar disorder. Schizophr. Bull..

[b0145] Jiang Y., Luo C., Li X., Li Y., Yang H., Li J., Chang X., Li H., Yang H., Wang J., Duan M., Yao D. (2019). White-matter functional networks changes in patients with schizophrenia. Neuroimage.

[b0150] Jones D.K. (2008). Tractography gone wild: probabilistic fibre tracking using the wild bootstrap with diffusion tensor MRI. IEEE Trans. Med. Imaging.

[b0155] Kai J., Khan A.R. (2022). Assessing the reliability of template-based clustering for tractography in healthy human adults. Front. Neuroinf..

[b0160] Kellner E., Dhital B., Kiselev V.G., Reisert M. (2016). Gibbs-ringing artifact removal based on local subvoxel-shifts. Magn. Reson. Med..

[b0165] Kelly S., Jahanshad N., Zalesky A., Kochunov P., Agartz I., Alloza C., Andreassen O.A., Arango C., Banaj N., Bouix S., Bousman C.A., Brouwer R.M., Bruggemann J., Bustillo J., Cahn W., Calhoun V., Cannon D., Carr V., Catts S., Chen J., Chen J.-x., Chen X., Chiapponi C., Cho K.K., Ciullo V., Corvin A.S., Crespo-Facorro B., Cropley V., De Rossi P., Diaz-Caneja C.M., Dickie E.W., Ehrlich S., Fan F.-m., Faskowitz J., Fatouros-Bergman H., Flyckt L., Ford J.M., Fouche J.-P., Fukunaga M., Gill M., Glahn D.C., Gollub R., Goudzwaard E.D., Guo H., Gur R.E., Gur R.C., Gurholt T.P., Hashimoto R., Hatton S.N., Henskens F.A., Hibar D.P., Hickie I.B., Hong L.E., Horacek J., Howells F.M., Hulshoff Pol H.E., Hyde C.L., Isaev D., Jablensky A., Jansen P.R., Janssen J., Jönsson E.G., Jung L.A., Kahn R.S., Kikinis Z., Liu K., Klauser P., Knöchel C., Kubicki M., Lagopoulos J., Langen C., Lawrie S., Lenroot R.K., Lim K.O., Lopez-Jaramillo C., Lyall A., Magnotta V., Mandl R.C.W., Mathalon D.H., McCarley R.W., McCarthy-Jones S., McDonald C., McEwen S., McIntosh A., Melicher T., Mesholam-Gately R.I., Michie P.T., Mowry B., Mueller B.A., Newell D.T., O'Donnell P., Oertel-Knöchel V., Oestreich L., Paciga S.A., Pantelis C., Pasternak O., Pearlson G., Pellicano G.R., Pereira A., Pineda Zapata J., Piras F., Potkin S.G., Preda A., Rasser P.E., Roalf D.R., Roiz R., Roos A., Rotenberg D., Satterthwaite T.D., Savadjiev P., Schall U., Scott R.J., Seal M.L., Seidman L.J., Shannon Weickert C., Whelan C.D., Shenton M.E., Kwon J.S., Spalletta G., Spaniel F., Sprooten E., Stäblein M., Stein D.J., Sundram S., Tan Y., Tan S., Tang S., Temmingh H.S., Westlye L.T., Tønnesen S., Tordesillas-Gutierrez D., Doan N.T., Vaidya J., van Haren N.E.M., Vargas C.D., Vecchio D., Velakoulis D., Voineskos A., Voyvodic J.Q., Wang Z., Wan P., Wei D., Weickert T.W., Whalley H., White T., Whitford T.J., Wojcik J.D., Xiang H., Xie Z., Yamamori H., Yang F., Yao N., Zhang G., Zhao J., van Erp T.G.M., Turner J., Thompson P.M., Donohoe G. (2018). Widespread white matter microstructural differences in schizophrenia across 4322 individuals: results from the ENIGMA Schizophrenia DTI Working Group. Mol. Psychiatry.

[b0170] Khan A., Hemachandra D., Kai J. (2021).

[b0175] Kraguljac N.V., White D.M., Hadley J.A., Visscher K., Knight D., ver Hoef L., Falola B., Lahti A.C. (2016). Abnormalities in large scale functional networks in unmedicated patients with schizophrenia and effects of risperidone. NeuroImage Clin..

[b0180] Limongi R., Jeon P., Mackinley M., Das T., Dempster K., Théberge J., Bartha R., Wong D., Palaniyappan L. (2020). Glutamate and dysconnection in the salience network: neurochemical, effective connectivity, and computational evidence in schizophrenia. Biol. Psychiatry.

[b0185] Lin C.-H., Lin H.-S., Lin S.-C., Kuo C.-C., Wang F.-C., Huang Y.-H. (2018). Early improvement in PANSS-30, PANSS-8, and PANSS-6 scores predicts ultimate response and remission during acute treatment of schizophrenia. Acta Psychiatr. Scand..

[b0190] Maas D.A., Vallès A., Martens G.J.M. (2017). Oxidative stress, prefrontal cortex hypomyelination and cognitive symptoms in schizophrenia. Transl. Psychiatry.

[b0195] MacKay A.L., Laule C., Zalc B. (2016). Magnetic resonance of myelin water: An in vivo marker for myelin. Brain Plast..

[b0200] Mäki P., Veijola J., Jones P.B., Murray G.K., Koponen H., Tienari P., Miettunen J., Tanskanen P., Wahlberg K.-E., Koskinen J., Lauronen E., Isohanni M. (2005). Predictors of schizophrenia—a review. Br. Med. Bull..

[b0205] Marques J.P., Kober T., Krueger G., van der Zwaag W., Van de Moortele P.-F., Gruetter R. (2010). MP2RAGE, a self bias-field corrected sequence for improved segmentation and T1-mapping at high field. Neuroimage.

[b0210] McIntosh A.M., Maniega S.M., Lymer G.K.S., McKirdy J., Hall J., Sussmann J.E.D., Bastin M.E., Clayden J.D., Johnstone E.C., Lawrie S.M. (2008). White matter tractography in bipolar disorder and schizophrenia. Biol. Psychiatry.

[b0215] Menon V., D’Esposito M. (2022). The role of PFC networks in cognitive control and executive function. Neuropsychopharmacology.

[b0220] Mighdoll M.I., Tao R., Kleinman J.E., Hyde T.M. (2015). Myelin, myelin-related disorders, and psychosis. Schizophr. Res..

[b0225] Mingoia G., Wagner G., Langbein K., Maitra R., Smesny S., Dietzek M., Burmeister H.P., Reichenbach J.R., Schlösser R.G.M., Gaser C., Sauer H., Nenadic I. (2012). Default mode network activity in schizophrenia studied at resting state using probabilistic ICA. Schizophr. Res..

[b0230] Morosini P.L., Magliano L., Brambilla L., Ugolini S., Pioli R. (2000). Development, reliability and acceptability of a new version of the DSM-IV Social and Occupational Functioning Assessment Scale (SOFAS) to assess routine social functioning. Acta Psychiatr. Scand..

[b0235] Mubarik A., Tohid H. (2016). Frontal lobe alterations in schizophrenia: a review. Trends Psychiatry Psychother..

[b0240] Nazeri A., Chakravarty M.M., Felsky D., Lobaugh N.J., Rajji T.K., Mulsant B.H., Voineskos A.N. (2013). Alterations of superficial white matter in schizophrenia and relationship to cognitive performance. Neuropsychopharmacology.

[b0245] Nichols T.E., Holmes A.P. (2002). Nonparametric permutation tests for functional neuroimaging: A primer with examples. Hum. Brain Mapp..

[b0250] O’Halloran R., Feldman R., Marcuse L., Fields M., Delman B., Frangou S., Balchandani P. (2017). A method for u-fiber quantification from 7 T diffusion-weighted MRI data tested in patients with nonlesional focal epilepsy. Neuroreport.

[b0255] Ouyang M., Kang H., Detre J.A., Roberts T.P.L., Huang H. (2017). Short-range connections in the developmental connectome during typical and atypical brain maturation. Neurosci. Biobehav. Rev..

[b0260] Phillips O.R., Nuechterlein K.H., Asarnow R.F., Clark K.A., Cabeen R., Yang Y., Woods R.P., Toga A.W., Narr K.L. (2011). Mapping corticocortical structural integrity in schizophrenia and effects of genetic liability. Biol. Psychiatry.

[b0265] Phillips O.R., Joshi S.H., Squitieri F., Sanchez-Castaneda C., Narr K., Shattuck D.W., Caltagirone C., Sabatini U., Di Paola M. (2016). Major superficial white matter abnormalities in Huntington’s disease. Front. Neurosci..

[b0270] Raffelt D., Tournier J.-D., Rose S., Ridgway G.R., Henderson R., Crozier S., Salvado O., Connelly A. (2012). Apparent Fibre Density: a novel measure for the analysis of diffusion-weighted magnetic resonance images. Neuroimage.

[b0275] Rubinov M., Bullmore Ed. 2013. Schizophrenia and abnormal brain network hubs. Dialogues Clin. Neurosci. 15:339–349. 10.31887/DCNS.2013.15.3/mrubinov PMC3811105 24174905

[b0280] Sarnat H.B., Hader W., Flores-Sarnat L., Bello-Espinosa L. (2018). Synaptic plexi of U-fibre layer beneath focal cortical dysplasias: Role in epileptic networks. Clin. Neuropathol..

[b0285] Schaefer A., Kong R., Gordon E.M., Laumann T.O., Zuo X.-N., Holmes A.J., Eickhoff S.B., Yeo B.T.T. (2018). Local-global parcellation of the human cerebral cortex from intrinsic functional connectivity MRI. Cereb. Cortex.

[b0290] Shastin D., Genc S., Parker G.D., Koller K., Tax C.M.W., Evans J., Hamandi K., Gray W.P., Jones D.K., Chamberland M. (2022). Surface-based tracking for short association fibre tractography. Neuroimage.

[b0295] Smith S.M., Jenkinson M., Woolrich M.W., Beckmann C.F., Behrens T.E.J., Johansen-Berg H., Bannister P.R, De Luca M., Drobnjak I., Flitney D.E., Niazy R.K., Saunders J., Vickers J., Zhang Y., De Stefano N., Brady J.M., Matthews P.M. 2004. Advances in functional and structural MR image analysis and implementation as FSL. NeuroImage 23 Suppl 1:S208-219. 10.1016/j.neuroimage.2004.07.051 15501092

[b0300] Smith R.E., Tournier J.-D., Calamante F., Connelly A. (2013). SIFT: Spherical-deconvolution informed filtering of tractograms. Neuroimage.

[b0305] Stark A.K., Uylings H.B.M., Sanz-Arigita E., Pakkenberg B. (2004). Glial cell loss in the anterior cingulate cortex, a subregion of the prefrontal cortex, in subjects with schizophrenia. Am. J. Psychiatry.

[b0310] Stephan K.E., Friston K.J., Frith C.D. (2009). Dysconnection in schizophrenia: from abnormal synaptic plasticity to failures of self-monitoring. Schizophr. Bull..

[b0315] Stilo S.A., Murray R.M. (2019). Non-genetic factors in schizophrenia. Curr. Psychiatry Rep..

[b0320] Sundaram S.K., Kumar A., Makki M.I., Behen M.E., Chugani H.T., Chugani D.C. (2008). Diffusion tensor imaging of frontal lobe in autism spectrum disorder. Cereb. Cortex.

[b0325] Thompson P.M., Schwartz C., Lin R.T., Khan A.A., Toga A.W. (1996). Three-dimensional statistical analysis of sulcal variability in the human brain. J. Neurosci..

[b0330] Tournier J.-D., Smith R., Raffelt D., Tabbara R., Dhollander T., Pietsch M., Christiaens D., Jeurissen B., Yeh C.-H., Connelly A. 2019. MRtrix3: A fast, flexible and open software framework for medical image processing and visualisation. NeuroImage 202:116137. 10.1016/j.neuroimage.2019.116137 31473352

[b0335] Uranova N.A., Vikhreva O.V., Rachmanova V.I., Orlovskaya D.D. 2011. Ultrastructural alterations of myelinated fibers and oligodendrocytes in the prefrontal cortex in schizophrenia: A postmortem morphometric study. Schizophr. Res. Treat. 2011:e325789. 10.1155/2011/325789 PMC3420756 22937264

[b0340] Veraart J., Sijbers J., Sunaert S., Leemans A., Jeurissen B. (2013). Weighted linear least squares estimation of diffusion MRI parameters: Strengths, limitations, and pitfalls. Neuroimage.

[b0345] Veraart J., Fieremans E., Novikov D.S. (2016). Diffusion MRI noise mapping using random matrix theory. Magn. Reson. Med..

[b0350] Veraart J., Novikov D.S., Christiaens D., Ades-aron B., Sijbers J., Fieremans E. (2016). Denoising of diffusion MRI using random matrix theory. Neuroimage.

[b0355] Vitolo E., Tatu M.K., Pignolo C., Cauda F., Costa T., Ando’ A., Zennaro A. (2017). White matter and schizophrenia: A meta-analysis of voxel-based morphometry and diffusion tensor imaging studies. Psychiatry Res. Neuroimaging.

[b0360] Wasserthal J., Maier-Hein K.H., Neher P.F., Northoff G., Kubera K.M., Fritze S., Harneit A., Geiger L.S., Tost H., Wolf R.C., Hirjak D. (2020). Multiparametric mapping of white matter microstructure in catatonia. Neuropsychopharmacology.

[b0365] Weinberger D.R., Berman K.F., Zec R.F. (1986). Physiologic dysfunction of dorsolateral prefrontal cortex in schizophrenia: I. Regional cerebral blood flow evidence. Arch. Gen. Psychiatry.

[b0370] Welch B.L. (1947). The generalization of “students” problem when seveveral difference population variances are involved. Biometrika.

[b0375] Wernicke C. (1906).

[b0380] Yoshino M., Saito K., Kawasaki K., Horiike T., Shinmyo Y., Kawasaki H. (2020). The origin and development of subcortical U-fibers in gyrencephalic ferrets. Mol. Brain.

[b0385] Zhang H., Schneider T., Wheeler-Kingshott C.A., Alexander D.C. (2012). NODDI: Practical in vivo neurite orientation dispersion and density imaging of the human brain. Neuroimage.

[bib387] Zhang F., Wu Y., Norton I., Rigolo L., Rathi Y., Makris N., O’Donnell L.J. (2018). An anatomically curated fiber clustering white matter atlas for consistent white matter tract parcellation across the lifespan. Neuroimage.

