# Supplementary material for: Aberrant frontal lobe “U”-shaped association fibers in first-episode schizophrenia: A 7-Tesla Diffusion Imaging Study
Source: Neuroimage Clin. 2023 Mar 5;38:103367. doi: 10.1016/j.nicl.2023.103367 (PMC10011060; doi:10.1016/j.nicl.2023.103367)
Supplement: Supplementary data 1 — Details the quantitative measures observed along the length identified aberrant tracts. [file mmc1.docx]

# Supplementary Materials - Analysis of affected segments

The following document provides a more in-depth statistical analysis of the segments demonstrating significant differences between controls and patients with first-episode schizophrenia (FES) along the length of identified aberrant tracts.



**Figure S1.** Along-tract measures for fractional anisotropy (FA) and radial diffusivity (RD) for controls and patients with FES. Segments exhibiting differences in both FA and RD are indicated by the number above, corresponding with segments from Table S1.

**Table S1.** Observed t-statistic and p-value from segments demonstrating differences from both along-tract FA and RD. Segment numbers correspond with the segments shown in Figure S1.

|  |  | **Cluster 9** | | **Cluster 46** | | **Cluster 65** | |
| --- | --- | --- | --- | --- | --- | --- | --- |
| **Metric** | **Segment** | t-stat | p-val | t-stat | p-val | t-stat | pval |
| FA | 1 | 3.337 | 0.00164 | 3.092 | 0.00323 | 3.600 | 0.00110 |
|  | 2 | 2.962 | 0.00472 |  |  | 3.443 | 0.00170 |
| RD | 1 | -3.638 | 0.000667 | -3.126 | 0.00273 | -3.284 | 0.00221 |
|  | 2 | -3.469 | 0.00113 |  |  | -3.555 | 0.000943 |
